# Mixed convective boundary layer flow over a vertical wedge embedded in a porous medium saturated with a nanofluid: Natural Convection Dominated Regime

**DOI:** 10.1186/1556-276X-6-207

**Published:** 2011-03-09

**Authors:** Rama Subba Reddy Gorla, Ali Jawad Chamkha, Ahmed Mohamed Rashad

**Affiliations:** 1 Cleveland State University, Cleveland, OH 44115 USA; 2 Manufacturing Engineering Department, The Public Authority for Applied Education and Training, Shuweikh 70654, Kuwait; 3 Department of Mathematics, Taibah University, Faculty of Science, Al Madina Al Munawara, Saudi Arabia; 4 Department of Mathematics, South Valley University, Faculty of science, Aswan, Egypt

## Abstract

A boundary layer analysis is presented for the mixed convection past a vertical wedge in a porous medium saturated with a nano fluid. The governing partial differential equations are transformed into a set of non-similar equations and solved numerically by an efficient, implicit, iterative, finite-difference method. A parametric study illustrating the influence of various physical parameters is performed. Numerical results for the velocity, temperature, and nanoparticles volume fraction profiles, as well as the friction factor, surface heat and mass transfer rates have been presented for parametric variations of the buoyancy ratio parameter *Nr*, Brownian motion parameter *Nb*, thermophoresis parameter *Nt*, and Lewis number *Le*. The dependency of the friction factor, surface heat transfer rate (Nusselt number), and mass transfer rate (Sherwood number) on these parameters has been discussed.

## Introduction

Nanofluids are prepared by dispersing solid nanoparticles in fluids such as water, oil, or ethylene glycol. These fluids represent an innovative way to increase thermal conductivity and, therefore, heat transfer. Unlike heat transfer in conventional fluids, the exceptionally high thermal conductivity of nanofluids provides for enhanced heat transfer rates, a unique feature of nanofluids. Advances in device miniaturization have necessitated heat transfer systems that are small in size, light mass, and high-performance. Several authors have tried to establish convective transport models for nanofluids. Nanofluid is a two-phase mixture in which the solid phase consists of nano-sized particles. In view of the nanoscale size of the particles, it may be questionable whether the theory of conventional two-phase flow can be applied in describing the flow characteristics of nanofluid. Nanofluids are also solid-liquid composite materials consisting of solid nanoparticles or nanofibers with sizes typically of 1-100 nm suspended in liquid. Nanofluids have attracted great interest recently because of reports of greatly enhanced thermal properties. For example, a small amount (<1% volume fraction) of Cu nanoparticles or carbon nanotubes dispersed in ethylene glycol or oil is reported to increase the inherently poor thermal conductivity of the liquid by 40 and 150%, respectively, as previously shown in [1,2]. Conventional particle-liquid suspensions require high concentrations (>10%) of particles to achieve such enhancement. However, problems of rheology and stability are amplified at high concentrations, precluding the widespread use of conventional slurries as heat transfer fluids. In some cases, the observed enhancement in thermal conductivity of nanofluids is orders of magnitude larger than that predicted by well-established theories. Other perplexing results in this rapidly evolving field include a surprisingly strong temperature dependence of the thermal conductivity [3] and a three-fold higher critical heat flux compared with the base fluids [4,5]. These enhanced thermal properties are not merely of academic interest. If confirmed and found consistent, then they would make nanofluids promising for applications in thermal management. Furthermore, suspensions of metal nanoparticles are also being developed for other purposes, such as medical applications including cancer therapy. The interdisciplinary nature of nanofluid research presents a great opportunity for exploration and discovery at the frontiers of nanotechnology. Porous media heat transfer problems have several engineering applications, such as geothermal energy recovery, crude oil extraction, ground water pollution, thermal energy storage, and flow through filtering media. Cheng and Minkowycz [6] presented similarity solutions for free convective heat transfer from a vertical plate in a fluid-saturated porous medium. Gorla and Tornabene [7] and Gorla and Zinolabedini [8] solved the nonsimilar problem of free convective heat transfer from a vertical plate embedded in a saturated porous medium with an arbitrarily varying surface temperature or heat flux. The problem of combined convection from vertical plates in porous media was studied by Minkowycz et al. [9], and Ranganathan and Viskanta [10]. Kumari and Gorla [11] presented an analysis for the combined convection along a non-isothermal wedge in a porous medium. All these studies were concerned with Newtonian fluid flows. The boundary layer flows in nano fluids have been analyzed recently by Nield and Kuznetsov and Kuznetsov [12] and Nield and Kuznetsov [13]. A clear picture about the nanofluid boundary layer flows is still to emerge.

This study has been undertaken to analyze the mixed convection past a vertical wedge embedded in a porous medium saturated by a nanofluid. The effects of Brownian motion and thermophoresis are included for the nanofluid. Numerical solutions of the boundary layer equations are obtained and discussion is provided for several values of the nanofluid parameters governing the problem.

### Analysis

**Figure 1:**
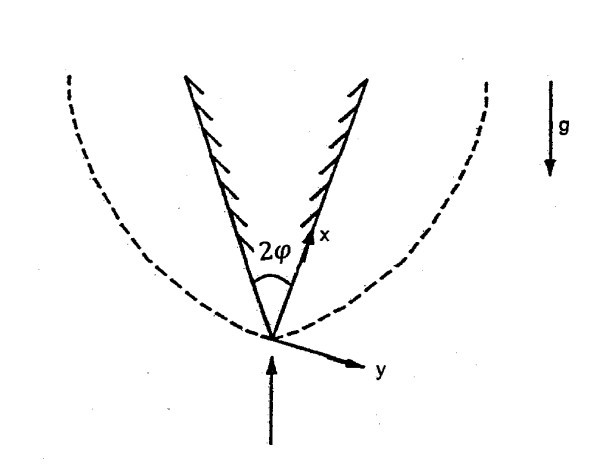
**Flow model and coordinate system**.

We consider the steady, free convection boundary layer flow past a vertical wedge placed in a nano-fluid-saturated porous medium. The co-ordinate system is selected such that *x*-axis is aligned with slant surface of the wedge. The flow model and coordinate system are shown in Figure 1.

We consider the two-dimensional problem. We consider at *y *= 0, the temperature *T *and the nano-particle fraction *ϕ *take constant values, *T*_W _and *ϕ*_W_, respectively. The ambient values, as *y *tends to infinity, of *T *and *ϕ *are denoted by *T*_∞ _and *ϕ*_∞_, respectively. The Oberbeck-Boussinesq approximation is employed. Homogeneity and local thermal equilibrium in the porous medium are assumed. We consider the porous medium whose porosity is denoted by *ε*, and permeability by *K*.

We now make the standard boundary layer approximation based on a scale analysis and write the governing equations.(1)(2)(3)(4)

where(5)

where, *ρ*_f_, *μ*, and *β *are the density, viscosity, and volumetric volume expansion coefficient of the fluid, while *ρ*_p _is the density of the particles. The gravitational acceleration is denoted by *g*. We have introduced the effective heat capacity (*ρc*)_m _and effective thermal conductivity, *k*_m_, of the porous medium. The coefficients that appear in Equations 3 and 4 are, respectively, the Brownian diffusion coefficient, *D*_B_, and the thermophoretic diffusion coefficient, *D*_T_.

The boundary conditions are taken to be(6)(7)

We introduce a stream line function ψ defined by(8)

so that Equation 1 is satisfied identically. We are then left with the following three equations:(9)(10)(11)

Proceeding with the analysis, we introduce the following dimensionless variables:(12)

Where *u*_∞ _= *cx*^m ^and *g*_*x *_= *g *cos *ϕ *represents the *x*-component of the acceleration due to gravity.

Substituting the expressions in Equation 12 into the governing Equations 9-11, we obtain the following transformed equations:(13)(14)(15)

where the parameters are defined as(16)

The transformed boundary conditions are(17)

It is noted that the *ξ *parameter here represents the forced flow effect on free convection. The case of *ξ *= 0 corresponds to pure free convection, and the limiting case of *ξ *= 1 corresponds to pure forced convection. The above system of Equations 13-15 was solved over the region covered by *ξ *= 0-1 to provide the other half of the solution for the entire mixed convection regime. Moreover, it may be remarked that the system of Equations 13-15 with the boundary conditions (17) reduces to the equations of combined convection along an isothermal wedge in a porous medium; when (*Nr *= *Nb *= *Nt *= 0), this case has been studied by Kumari and Gorla [11].

The local friction factor is given by(18)

The heat transfer rate is given by(19)

The heat transfer coefficient is given by(20)

Local Nusselt number is given by(21)

The mass transfer rate is given by(22)

where *h*_m _= mass transfer coefficient,(23)

and Sherwood number is given by(24)

## Numerical Method and Validation

Equations 13-15 represent an initial-value problem with *ξ *playing the role of time. This general non-linear problem cannot be solved in closed form and, therefore, a numerical solution is necessary to describe the physics of the problem. The implicit, tridiagonal finite-difference method similar to that discussed by Blottner [14] has proven to be adequate and sufficiently accurate for the solution of this kind of problems. Therefore, it is adopted in the present study. All the first-order derivatives with respect to *ξ *are replaced by two-point backward-difference formulae when marching in the positive *ξ *direction. Then, all the second-order differential equations in η are discretized using three-point central difference quotients. This discretization process produces a tri-diagonal set of algebraic equations at each line of constant *ξ *which is readily solved by the well-known Thomas algorithm (see Blottner [14]). During the solution, iteration is employed to deal with the nonlinearity aspect of the governing differential equations. The problem is solved line by line starting with line *ξ *= 0 where similarity equations are solved to obtain the initial profiles of velocity, temperature and concentration, and marching forward (or backward) in *ξ *until the desired line of constant *ξ *is reached. Variable step sizes in the η direction with Δη_1 _= 0.001 and a growth factor *G *= 1.035 such that Δη_*n *_= *G*Δη_*n*__-1 _and constant step sizes in the *ξ *direction with Δ*ξ *= 0.01 are employed. These step sizes are arrived at after many numerical experimentations performed to assess grid independence. The convergence criterion employed in this study is based on the difference between the current and the previous iterations. When this difference reached 10^-5 ^for all the points in the ηdirections, the solution was assumed to be converged, and the iteration process was terminated.

## Results and discussion

In this section, a representative set of graphical results for the dimensionless velocity *S *'(*ξ*,*η*), temperature *θ*(*ξ*,*η*), and nano-particle volume fraction *f*(*ξ*,*η*) as well as the local skin-friction coefficient *C*_f__*x *_= *S*"(*ξ*,0) (reciprocal of local friction factor), reduced local Nusselt number *Nu*_*x *_= -*θ*"(*ξ*,0) (reciprocal of rate of heat transfer), and the reduced local Sherwood number *Sh*_*x *_= -*f*"(*ξ*,0) (reciprocal of rate of mass transfer) is presented and discussed for various parametric conditions. These conditions are intended for various values of buoyancy ratio, *Nr*, Lewis number *Le*, thermophoresis parameter *Nt*, Brownian motion parameter *Nb*, wedge angle parameter m, and mixed convection parameter *ξ*, respectively.

**Figure 2:**
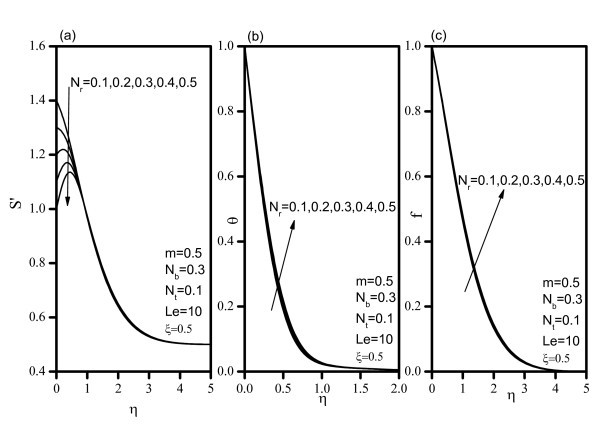
**Velocity, temperature, and concentration profiles for various values of Buoyancy Ratio *(Nr)***.

**Figure 3:**
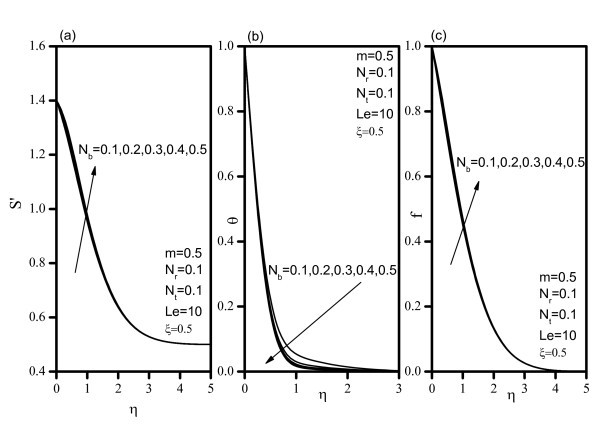
**Velocity, temperature, and concentration profiles for various values of Brownian motion parameter *(Nb)***.

**Figure 4:**
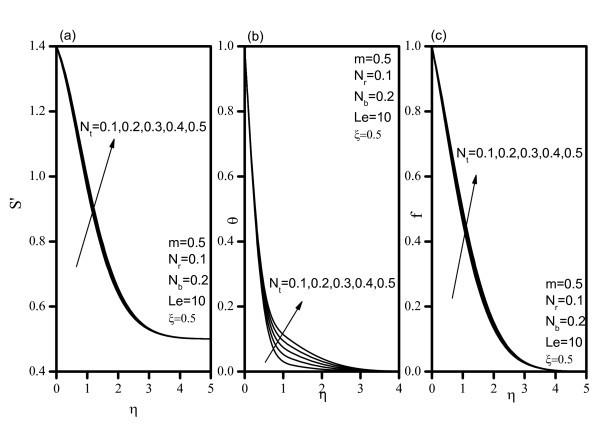
**Velocity, temperature, and concentration profiles for various values of Thermophoresis parameter *(Nt)***.

**Figure 5:**
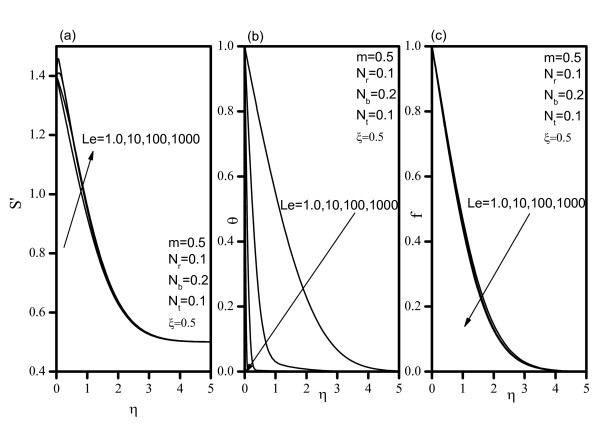
**Velocity, temperature, and concentration profiles for various values of Lewis number *(Le)***.

**Figure 6:**
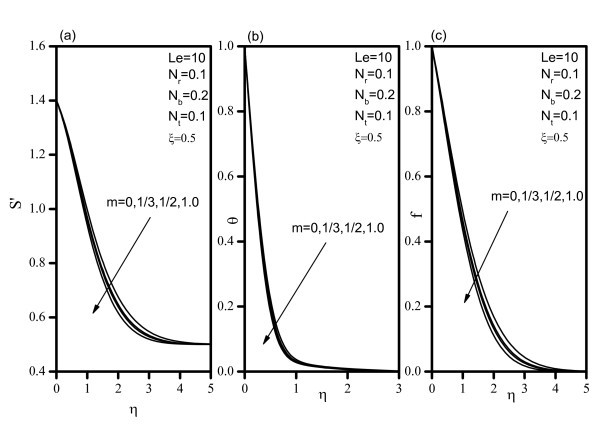
**Velocity, temperature, and concentration profiles for various values of velocity exponent *(m)***.

Figure 2 indicates that, as *Nr *increases, the velocity decreases, and the temperature and concentration increase. Similar effects are observed from Figures 3 and 4 as *Nt *and *Nb *vary. Figure 5 illustrates the variation of velocity within the boundary layer as *Le *increases. The velocity increases as *Le *increases. As *Le *increases, the temperature and concentration within the boundary layer decrease and the thermal and concentration boundary later thicknesses decrease. Figure 6 shows that as the wedge angle parameter m increases, the velocity, temperature, and concentration decrease.

**Figure 7:**
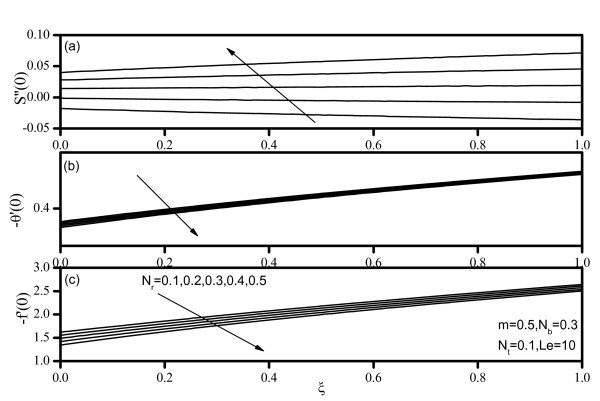
**Friction factor, Nusselt number, and Sherwood number for various values of Buoyancy Ratio *(Nr)***.

**Figure 8:**
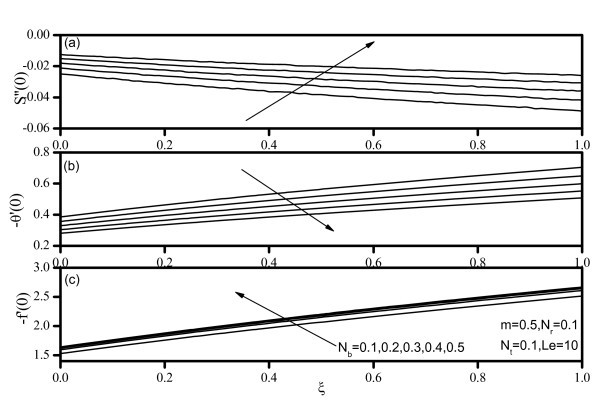
**Friction factor, Nusselt number, and Sherwood number for various values of Brownian motion parameter *(Nb)***.

**Figure 9:**
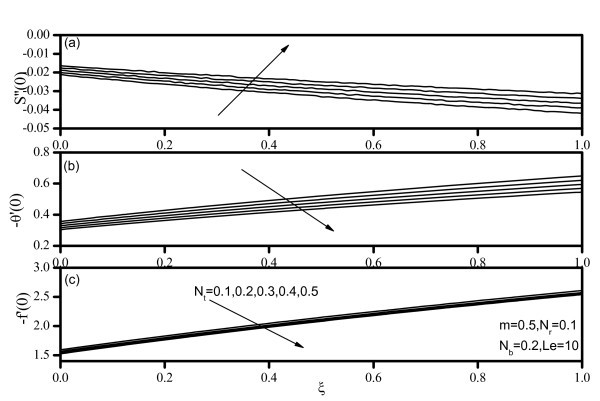
**Friction factor, Nusselt number, and Sherwood number for various values of Thermophoresis parameter *(Nt)***.

**Figure 10:**
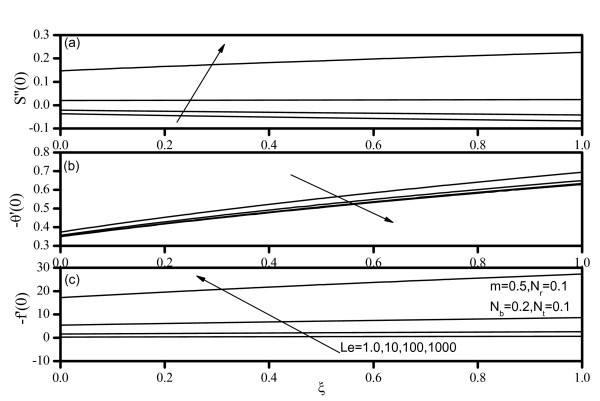
**Friction factor, Nusselt number, and Sherwood number for various values of Lewis number *(Le)***.

**Figure 11:**
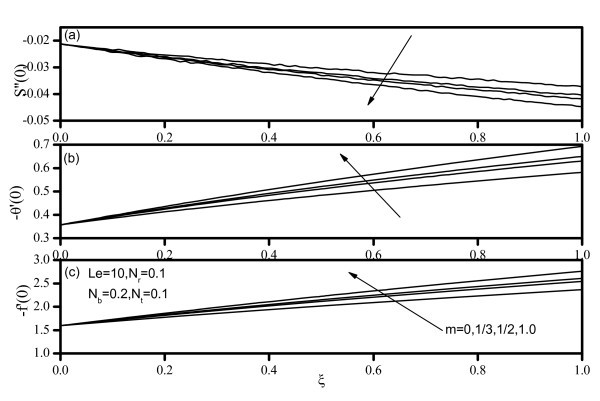
**Friction factor, Nusselt number, and Sherwood number for various values of velocity exponent *(m)***.

Figures 7, 8, 9, 10, and 11 display results for wall values for the gradients of velocity, temperature, and concentration functions which are proportional to the friction factor, Nusselt number, and Sherwood number, respectively. From Figures 7 and 9, we notice that as *Nr *and *Nt *increase, the friction factor increases whereas the heat transfer rate (Nusselt number) and mass transfer rate (Sherwood number) decrease. As *Nb *increases, the friction factor and surface mass transfer rates increase whereas the surface heat transfer rate decreases as shown by Figure 8. Figure 10 indicates that as *Le *increases, the heat transfer rate decreases whereas the mass transfer rate increases. From Figure 11, we observe that, as the wedge angle parameter m increases, the heat and mass transfer rates increase.

## Concluding Remarks

In this article, we presented a boundary layer analysis for the mixed convection past a vertical wedge embedded in a porous medium saturated with a nano fluid. Numerical results for friction factor, surface heat transfer rate, and mass transfer rate have been presented for parametric variations of the buoyancy ratio parameter *Nr*, Brownian motion parameter *Nb*, thermophoresis parameter *Nt*, and Lewis number *Le*. The results indicate that, as *Nr *and *Nt *increase, the friction factor increases, whereas the heat transfer rate (Nusselt number) and mass transfer rate (Sherwood number) decrease. As *Nb *increases, the friction factor and surface mass transfer rates increase, whereas the surface heat transfer rate decreases. As *Le *increases, the heat transfer rate decreases, whereas the mass transfer rate increases. As the wedge angle increases, the heat and mass transfer rates increase.

## Abbreviations

List of symbols

*D*_B_: Brownian diffusion coefficient; *D*_T_: Thermophoretic diffusion coefficient; *f*: Rescaled nano-particle volume fraction; *g*: Gravitational acceleration vector; *k*_m_: Effective thermal conductivity of the porous medium; *K*: Permeability of porous medium; *Le*: Lewis number; *Nr*: Buoyancy Ratio; *Nb*: Brownian motion parameter; *Nt*: Thermophoresis parameter; *Nu*: Nusselt number; *P*: Pressure; *q*": Wall heat flux; *Ra*_*x*_: Local Rayleigh number; *r*: Radial coordinate from the center of the wedge; *S*: Dimensionless stream function; *T*: Temperature; *T*_W_: Wall temperature at vertical wedge; *T*_∞_: Ambient temperature attained as y tends to infinity; *U*: Reference velocity; *u*, *v*: Velocity components; (*x*, *y*): Cartesian coordinates

Greek symbols

*α*_m_: Thermal diffusivity of porous medium; *β*: Volumetric expansion coefficient of fluid; *ε*: Porosity; *η*: Dimensionless distance; *θ*: Dimensionless temperature; *μ*: Viscosity of fluid; *ρ*_f_: Fluid density; *ρ*_p_: Nano-particle mass density; (*ρc*)_f_: Heat capacity of the fluid; (*ρc*)_m_: Effective heat capacity of porous medium; (*ρc*)_p_: Effective heat capacity of nano-particle material; τ: Parameter defined by equation (13); *ϕ*: Nano-particle volume fraction; *ϕ*_W_: Nano-particle volume fraction at vertical wedge; *ϕ*_∞_: Ambient nano-particle volume fraction attained; *ψ*: Stream function

## Competing interests

The authors declare that they have no competing interests.

## Authors' contributions

RSRG conceived of the research and formulated the analysis, derived all the equations and wrote the paper. AJC contributed with the numerical solution of the governing transformed equations. AMR helped with a portion of the numerical analysis, and preparation of figures. All authors read and approved the final manuscript.
